# Effect of Organic Acid-Aided Extraction on Characteristics and Functional Properties of Pectin from *Cannabis sativa* L.

**DOI:** 10.3390/molecules29112511

**Published:** 2024-05-26

**Authors:** Nopparat Prabsangob, Sasithorn Hangsalad, Thepkunya Harnsilawat

**Affiliations:** Department of Product Development, Faculty of Agro-Industry, Kasetsart University, Bangkok 10900, Thailand

**Keywords:** cannabis, pectin, emulsifying property, antioxidant activity, foaming property

## Abstract

The extraction of cannabinoids from the inflorescence and leaves of *Cannabis sativa* L. is gaining interest from researchers, in addition to addressing the under-utilization of the by-products in the stems and roots of the trees. The present study investigated the recovery of pectin from the left-over parts of hemp tress using an eco-friendly method with the aid of organic acids. Different cannabis cultivars—Chalotte’s Angels (CHA) and Hang-Krarog (HKR)—were used as plant materials. The stems of both cannabis cultivars contained more pectin than the roots, and tartaric acid-aided extraction provided higher yields than from citric acid. Extracting the acid solution affected some characteristics, thereby differentiating the functional properties of the derived pectin. Extraction using tartaric acid provided pectin with a higher galacturonic acid content, whereas pectin with a higher methylation degree could be prepared using citric acid. The pectin samples extracted from the stems of CHA (P-CHA) and HKR (P-HKR) had low methoxyl pectin. P-CHA had better free radical scavenging capability, whereas P-HKR showed more potent reducibility. Considering the functional properties, P-CHA showed greater emulsion formability and foaming activity, whereas P-HKR possessed a better thickening effect. The present work suggests the feasible utilization of P-CHA and P-HKR as food additives with bioactivity.

## 1. Introduction

Pectin is a functional polysaccharide widely used in the production of foods and pharmaceuticals, owing to several functional properties, particularly its gelling, thickening, and emulsifying abilities. In addition, pectin possesses some bioactivities, such as antioxidant, anticancer, and hypoglycemic capacities [1,2], making it an interesting candidate for functional food development. Pectin is a complex polysaccharide that is naturally present as an adhesive agent in plant cell walls. Pectin consists of a galacturonic acid backbone, covalently linked with a sidechain of other monosaccharides, such as rhamnose, glucose, and galactose [1,3]. Crucially, both intrinsic (variety of plant) and extrinsic (extraction method) factors affect the molecular characteristics of pectin, thereby determining dissimilar functional properties between different sources of pectin and the extraction method. Pectin is generally classified into two types according to the degree of methylation (DM)—high methoxyl pectin (HMP) and low methoxyl pectin (LMP)—having DM values higher than 50% and less than 50%, respectively [4]. The gelation of HMP always requires a high sugar content (ca. 65%) or another co-solute to facilitate the formation of hydrogen bonds and the hydrophobic interaction of structural methoxyl groups [5]. This may limit the use of HMP in some foods, particularly for products with low-calorie and dietetic effects. On the other hand, the gelation of LMP occurs mainly through the ion mediation of divalent cations that can occur over a wide pH range with no sugar requirement [6].

Commercial pectin is generally produced from citrus peel and apple pomace through acid-aided extraction, with inorganic acids typically used for this purpose, such as nitric, sulfuric, and hydrochloric acids [2,7]. These mineral acids may cause toxicity and not be environmentally friendly. Nowadays, green extraction is an interesting global trend to support sustainability. Organic acids, such as citric and tartaric acids, are interesting alternatives to harsh mineral acids, owing to the former being safe and not harmful to the environment [8]. It has been reported that citric and tartaric acids could be applied as the extracting solvent to recover pectin from the peels of several plants, such as jackfruit [8], potato [9], and apple [10].

*Cannabis sativa* L. is a fibrous annual plant with a stem diameter of ca. 4–20 mm [11]. *C. sativa* is used in the production of food and feed, as well as for therapeutic purposes due to the presence of several bioactive compounds, especially the cannabinoids Δ9-tetrahydrocannabinol (THC) and cannabidiol (CBD) [12]. These cannabinoids can react with the receptors of the endocannabinoid system, resulting in the ability to control some responses from the central and peripheral nervous systems, such as pain, muscle relaxation, hunger, and nausea [13]. The cannabinoids are most abundant in the inflorescence and leaves of cannabis trees [14]. Preparation of the cannabinoids as bioactive compounds results in some left-over parts of the cannabis tree that are under-utilized material, including roots and stems. Considering cannabis use for edible purposes, the excess consumption of cannabinoids must be considered because of their side effects, such as a change in physiological parameters (blood pressure, heart rate, and body temperature), drowsiness, anesthesia, and anorexia [15]. Although CBD and CBD-infused food products are commercially available, the daily intake of more than 2 mg/kg CBD is suggested to be unsafe [16]. The stems and roots of *C. sativa* possess very low cannabinoids, with the contents of 0.1–0.4 mg/kg and 0.5–0.8 mg/kg of plant materials, respectively [17]. Therefore, these under-utilized materials may be a practical source of pectin to be used as food additives.

The present work aimed to extract pectin from the left-over parts of the cannabis tree through an eco-friendly method using organic acid. Food-grade organic acids (citric and tartaric) were used as separate extracting solvents due to their approved GRAS status (generally recognized as safe). The characteristics, functional properties, and antioxidant activity of the extracted pectin samples were examined to elucidate the feasible use of the extracted pectin as a food additive with health-promoting effects.

## 2. Results and Discussion

### 2.1. Effects of Extracting Acid Solutions on Physiochemical Properties of Cannabis Pectin

**Figure 1:**
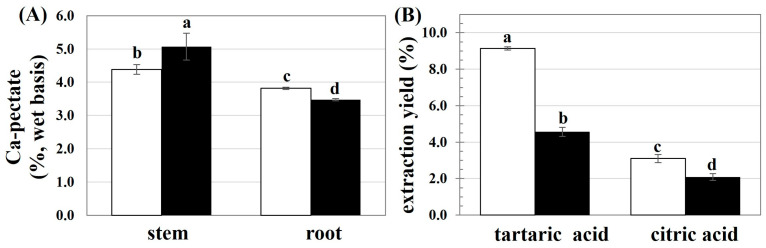
(**A**) Calcium pectate content present in selected parts of CHA (□) and HKR (■) and (**B**) yield of pectin extracted from stems of CHA (□) and HKR (■) using different acid solutions. In each subfigure, different letters indicate significant differences between means (*p* ≤ 0.05).

First, the availability of pectin in the stems and roots of the CHA and HKR cannabis trees was pre-estimated as the calcium pectate content, with the result shown in Figure 1A. There was a higher calcium pectate content in the stems of both cannabis cultivars. Cannabis is a crop with phenotypic variety in morphology and chemical composition [12]. Both intrinsic (accession and part plants) and extrinsic (growing conditions) factors have been reported to affect the chemical composition of cannabis fibers [12]. The main components of the cannabis bast fibers are cellulose, hemicellulose, lignin, and pectin [18]. Most of the pectin is present in the middle lamellae between plant cells and is involved in intracellular adhesion, thereby playing an important role in the integrity and rigidity of plant tissues [12]. In addition, pectin plays a crucial role in defending photogenic microorganisms [12], regulating ion transport, and providing water holding ability [19] for plants. In the present study, due to their higher contents of calcium pectate, the stems of HKR and CHA were used as raw materials for further study.

The extraction of pectin from the stems of CHA and HKR was performed using tartaric and citric acid solutions, with the extraction yields shown in Figure 1B. The extracting solvent greatly affected the yield of pectin, with a higher extraction yield achieved using tartaric acid than citric acid. The type and concentration of the acid crucially affect the pectin extraction efficiency [8,9]. Tartaric acid possesses two terminal carboxylic groups, whereas citric acid has triple-terminal carboxylic groups; consequently, higher hydrolytic power could be expected for citric acid [20]. The lower hydrolyzing ability of the acid solution might have less of an effect on the acidolysis degradation of the extracted pectin [8,21]. Therefore, a higher extractable pectin yield might be expected for extraction using tartaric acid compared to citric acid. Effective pectin recovery from passion fruit peels was also reported using tartaric acid as the extracting solvent than a stronger acid solution such as nitric acid [20]. In another study [10], there was a higher yield of pectin extracted from apple peel using tartaric acid (6.2%) compared to citric acid (5.3%).

The effect of acid solution on the characteristics of the recovered pectin was investigated. The contents of GalA and methoxyl groups in the derived pectin are presented in Table 1. Extraction using tartaric acid produced pectin with a higher GalA content compared to that recovered using citric acid. Lower GalA content was also reported for hawthorn pectin prepared using an acid solution (HCl) with higher hydrolytic power compared to one with lower hydrolytic power such as citric acid [2]. Dissolution of the non-pectin ingredients could be expected when an acid with strong hydrolytic power is used, thereby lowering the GalA content of the derived pectin [22]. In the present study, a higher GalA content was achieved for the pectin extracted from the stems of CHA compared to that derived from the stems of HKR, implying greater linearity of the pectin derived from the stems of CHA [23]. The accession of plants is an important intrinsic factor affecting the chemical structure present in dissimilar GalA contents in the pectin [12]. Based on the FAO and EU guidelines, the GalA content of pectin should not be lower than 65% [24], suggesting the pectin extracted in the present study might be utilized as a food additive.

The cannabis variety and extracting solvent seemed to have no effect on the methoxyl content. Methoxyl residues are reportedly related to functional properties, which impact the gelation behavior of pectin [5,6]. The methoxyl content of the pectin extracted in the present study was in a range of ca. 5.9–6.6%, implying that the pectin might be LMP. A comparable range of methoxyl contents has been reported for the LMP pectin recovered from other plant materials, such as lemon pomace (4.3–10.3%) [25], mango peel (7%), pomelo peel (8.6%), and lime residue (10%) [26].

**Figure 2:**
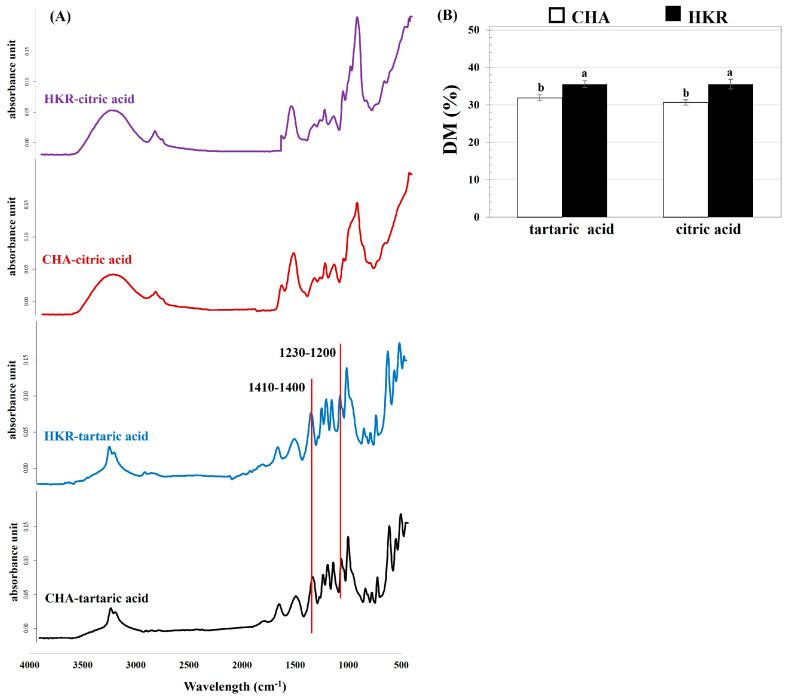
(**A**) FT-IR patterns and (**B**) DM of pectin extracted from the stems of CHA and HMR using different acid solutions. In subfigure (**B**), different letters indicate significant differences as affected by cannabis cultivar (*p* ≤ 0.05). Extracting acid solution had no significant effect on DM of derived pectin (*p* > 0.05).

The chemical structure of the derived pectin was determined, and the FT-IR profile of the pectin is depicted in Figure 2. Typical FT-IR spectra relating to pectin were observed, which correspond to the pectin extracted from other plant materials such as hawthorn wine pomace [2] and eggplant peel [23]. The typical FT-IR spectra for pectin involve several peaks: ca. 3600–3200 cm^−1^ relating to O-H stretching vibration of the intra– and inter-molecular hydrogen bonds of galacturonic units; ca. 3000–2800 cm^−1^ correlating with C-H stretching typically including CH, CH_2_, and CH_3_ stretching and bending vibrations; ca. 1750–1730 cm^−1^ for methyl-esterified carboxyl groups (COO-R)], ca. 1650–1600 cm^−1^ and 1410–1400 cm^−1^ for asymmetric and symmetric stretching modes of the carboxylated groups, respectively; ca. 1330–1320 cm^−1^ related to the C-H vibration of the pyranose ring; and ca. 1200–900 cm^−1,^ representing skeletal C-O and the C-C vibration of the glycosidic and pyranose linkages [27]. 

Extraction using different acid solutions affected the FT-IR profile of the derived pectin. Compared to the pectin recovered using citric acid, there were some peaks predominantly found for the pectin extracted using tartaric acid, particularly the peaks at 1410–1400 cm^−1^ and 1230–1200 cm^−1^. Greater areas for the peaks at ca. 1410–1400 cm^−1^ and 1230–1200 cm^−1^ were reported for pectin with a higher GalA content [28]. Moreover, the thinner absorption band in the region of 3500–3000 cm^−1^ of the pectin derived from tartaric acid-aided extraction might be attributed to the higher GalA amount of pectin [29]. This trend agreed with the higher GalA content of the pectin extracted using tartaric acid, as reported in the present study (Table 1).

The DM of the pectin was quantified using the FT-IR profile, with the results shown in Figure 2B. The DM (indicating the proportion of GalA units, which are methyl-esterified at C-6 to the total GalA units of pectin) is an important factor related to the functional properties of pectin such as the gelation ability and bioactivity [8]. The present results indicate that the pectin derived from the stems of both CHA and HKR was LMP, with a DM of ca. 30–35%. The extracting acid solution seemed to have no effect on the DM of the derived pectin. This might have been due to controlling the pH (pH 2.0) during the pectin recovery process, irrespective of the extracting acid solution. It has been suggested that the pH conditions played a crucial role in the DM of the extracted pectin [7]. The pectin derived from the stems of HKR had a higher DM than from the CHA stems, implying a greater degree of branching in the former pectin [30]. This trend agreed with the greater linearity of the pectin extracted from the CHA stems, as previously implied (Table 1). The DM (ca. 30–35%) and methoxyl content (ca. 6–7%) of the presently derived pectin were in in a comparable range with the LMP extracted from other plant materials, such as dragon fruit peels (DM of ca. 31–47% and methoxyl content of ca. 3%) [31] and over-ripened lemon pomace (DM of ca. 34% and methoxyl content of ca. 4%) [25]. In general, commercial pectin is extracted from apple pomace and citrus peel and it is always HMP with a DM higher than 70% [4]. On the other hand, LMP is present only in a few agricultural products, such as sunflower seeds and rods [32] and *Cyclea barbata* Miers [33] with a low yield, so commercial LMP is usually produced by modifying HMP through chemical- (acid, alkali, and ammonia in alcoholic media) and enzymatic- (pectin methyl esterase) assisted processes [5]. Therefore, the stems of CHA and HKR might be an interesting alternative source for LMP production.

**Figure 3:**
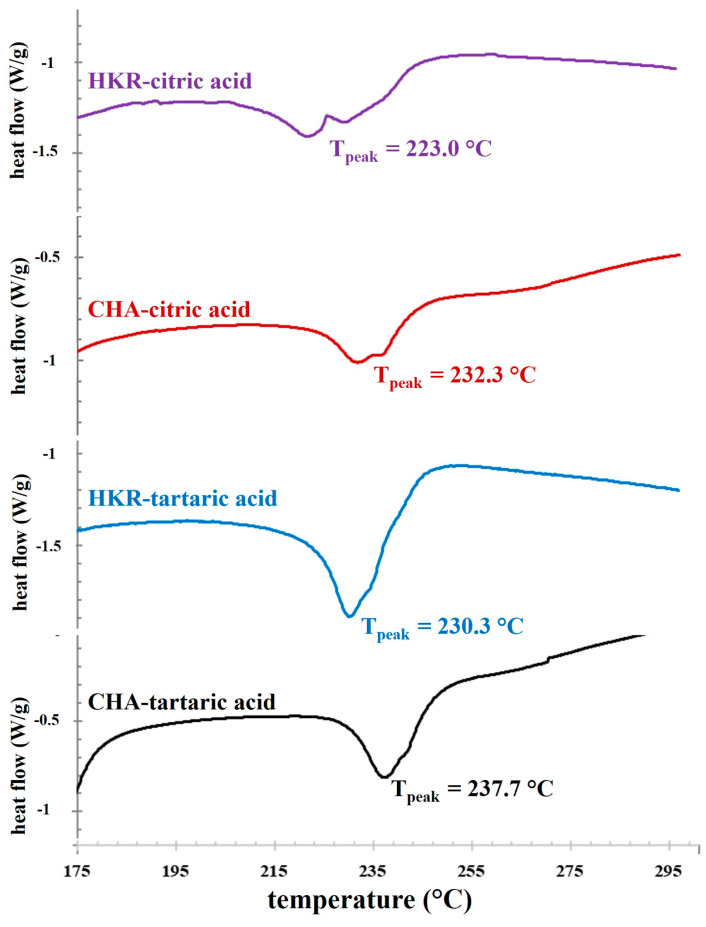
DSC thermograms of pectin extracted from the stems of CHA and HMR using different acid solutions.

Next, the thermal properties of the extracted pectin were determined using a DSC technique, and a DSC thermogram of the pectin samples is illustrated in Figure 3. A distinctive thermal event was observed as the exothermic peaks with thermal transition midpoints (T_peak_) of ca. 223–238 °C. These exothermic peaks might be related to a the degradation temperature of pectin [2]. Pectin typically possesses an exothermic degradation peak with dissimilar T_peak_ depending on plant sources, such as 245.5 °C for apple pomace pectin [34] and 233.8–246.8 °C for citrus peel pectin [35]. The present result indicates that the cannabis cultivars and extracting acid solution influenced the T_peak_ of the exothermic peaks of the derived pectin. Higher T_peak_ tended to be found for pectin extracted using tartaric acid compared to citric acid, suggesting better thermal stability for pectin extracted using tartaric acid [36]. Moreover, the pectin derived from CHA possessed higher T_peak_ than the counterparts recovered from HKR. This behavior might be expected due to the different chemical characteristics of the pectin recovered from dissimilar plant cultivars using different extracting acid solutions. The previous result indicated that extraction using tartaric acid provided pectin with a higher GalA content (Table 1), suggesting greater linearity in the pectin. With a lower degree of branching, the inter- and intra-attractive forces between the pectin chains were favorable due to the elimination of the repulsive force among the negatively charged carboxyl residues (-COO^−^) and a reduced steric hindrance effect of the side chains, so a more compact structure could be expected, resulting in better thermal stability in the pectin extracted using tartaric acid [35,37]. By using the corresponding extracting acid solution, greater thermal stability of the pectin derived from CHA was also coincidental with its higher GalA content, as compared to the ones recovered from HKR (Table 1). 

The present results indicate that the extraction using tartaric acid provided pectin with a higher extraction yield and better thermal stability. Pectin with greater thermal stability might be effectively utilized in some foods using a high processing temperature such as baked products [26]. Therefore, extraction using tartaric acid solution was performed to prepare pectin from the stems of CHA and HKR for further study, with the derived pectin samples referred to as P-CHA and P-HKR, respectively.

### 2.2. Characteristics and Functional Properties of P-CHA and P-HKR

The monosaccharide compositions of P-CHA and P-HKR were determined, and the results are shown in Table 2. Generally, pectin consists of four domains joined together by covalent interaction: linear homo-galacturonan (HG), rhamnogalacturonan I (RG-I), rhamnogalacturonan II (RG-II), and xylogalacturonan [3]. The HG domain mainly consists of an α-(1,4)-D-galacturonic acid pyranose residue, which is partially methoxylated [1]. The RG-I region contains a repeating unit of α-(1,4)-D-galacturonic acid pyranose and rhamno-pyranose as a backbone, which is esterified with the side chains of neutral monosaccharides such as arabinose and galactose [38]. The RG-II region has an HG backbone linked together with oligosaccharide chains of rare sugar moieties, such as fucopyranose, xylopyranose, apinofuranose, and xylofuranose [1,3]. In the xylogalacturonan region, the major monosaccharides are α-(1,4)-D-galacturonic acid pyranose and xylopyranose [3].

The predominant monosaccharides of P-CHA and P-HKR were GalA and glucuronic acid (GlcA) with the presence of others—rhamnose (Rha), arabinose (Ara), galactose (Gal), and glucose (Glu). The availability of Glu might be due to the presence of non-pectic polysaccharides, such as cellulose and hemicellulose, which were bound to the pectin in the plant cellular matrix [29]. P-CHA had a higher GalA content than P-HKR, indicating a greater presence of the linear HG backbone in P-CHA. This trend suggests a lower degree of branching for P-CHA [29], which was correlated with the lower DM of P-CHA compared to P-HKR, as shown in Figure 2B. Typically, GalA is abundantly present in the HG and RG-I domains of pectin [8,39]. In the present study, the Rha/GalA ratios of P-CHA and P-HKR were in a range of 0.05–1, suggesting that they consisted predominantly of RG-I regions [40]. Pectin with a high RG-I domain has a potent emulsifying property [41] and bioactivity to inhibit cardiovascular disease, cancer, and fibrosis [42].

The P-CHA and P-HKR contained GlcA, which is a rare compound usually found in pectin extracted from specific plant sources such as the flowers of *Tilia tomentosa* [39] and Kiwano (*Cucumis metuliferus*) peels [43]. Notably, the glucuronidated pectin extracted from the *T. tomentosa* flowers and Kiwano peels exhibited potent bioactivity to modulate and enhance immune activity [39,43]. In addition, P-HKR possessed Fuc that might imply the availability of the RG-II domain in pectin [29]. Fuc is a rare sugar, which is always found in the RG-II region and might be slightly present at the ends of the monosaccharide branches of the RG-I domain of pectin [44]. Effective anti-proliferation activity against Caco-2 cells was reported for the RG-II fraction of citrus pectin [1]. In addition, it has been reported that the rich polysaccharides in Fuc could provide antitumor effects [45]. The present results suggest that P-CHA and P-HKR might possess bioactivity, and this should be verified using further testing.

**Figure 4:**
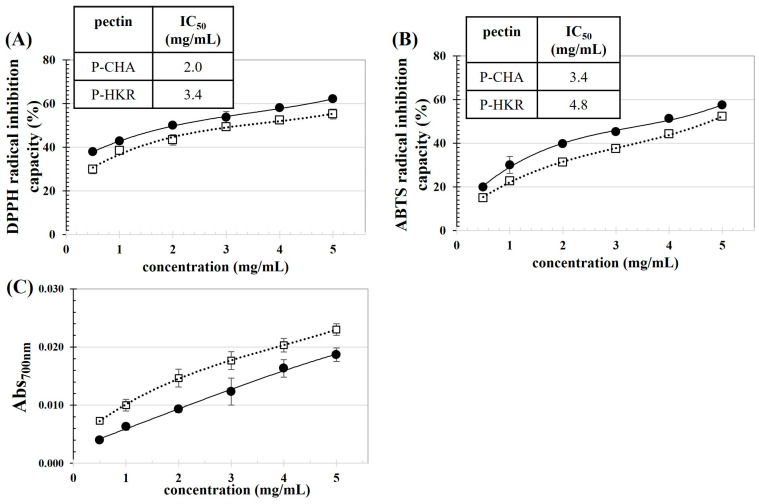
Concentration dependence on antioxidant activities of P-CHA (●) and P-HKR (□): (**A**) DPPH radical inhibition capacity, (**B**) ABTS radical inhibition capacity, and (**C**) reducibility. The inset tables in subfigures (**A**,**B**) represent concentrations of the pectin to scavenge 50% of DPPH and ABTS radicals (IC_50_).

The antioxidant activity of P-CHA and P-HKR was examined, as reported in Figure 4. P-CHA (15.38 ± 0.17 mg gallic acid equivalent/g pectin) had a higher TPC than P-HKR (13.88 ± 0.34 mg gallic acid equivalent/g pectin). In the plant cellular matrix, some parts of the phenolics are bound tightly to pectin, thereby eluting and co-precipitating with the extracted pectin [46]. The binding of phenolic acids to pectin occurs preferably at the arabinan and galactan chains of the RG-I fraction of pectin [19]. P-CHA had higher levels of GalA, Ara, and Gal residues than P-HKR, suggesting higher HG and RG-I domains in P-CHA. This might explain the higher TPC of P-CHA compared to P-HKR.

There was a positive relationship between the pectin concentration and antioxidant capacity for all observed parameters, including DPPH and ABTS radical scavenging abilities and reducibility. This behavior showed the same trend as the pectin derived from other plant sources such as apple pomace [46] and sunflower residues [47]. Greater DPPH and ABTS radical scavenging abilities were found for P-CHA than P-HKR, which might have been due to the higher TPC of P-CHA. A good correlation between TPC and DPPH and ABTS radical scavenging ability has been reported for apple pomace pectin [46]. It has been reported that GalA could exhibit free radical scavenging ability [48]; thus, more efficient free radical scavenging abilities might be supposed for P-CHA with its higher GalA content [47]. From the present results, the IC_50_ values to scavenge the DPPH radicals (ABTS radicals) of P-CHA and P-HKR were ca. 2.0 mg/mL (3.4 mg/mL) and 3.4 mg/mL (4.8 mg/mL), respectively. The IC_50_ against ABTS radicals of the pectin derived from citrus peels has been reported in a range of 2.1–9.1 mg/mL [35].

In contrast, with reducibility, there was greater activity for P-HKR than P-CHA. This might be due to the different modes of action of the phenolics present, as well as the chemical characteristics of the pectin sample. P-HKR contained Fuc, which might provide effective reducibility. The rich contents of polysaccharides in the Fuc recovered from brown seaweed (*Lobophora variegate)* provided potent reducibility with iron chelating activity up to 85%, whereas its phenolic content was very low [45]. Chemical characteristics, such as composited monosaccharides, the type of glycosidic linkages, as well as the presence of proteins, could affect the antioxidant capacity and antioxidant modes of action of polysaccharides [35,46,47].

**Figure 5:**
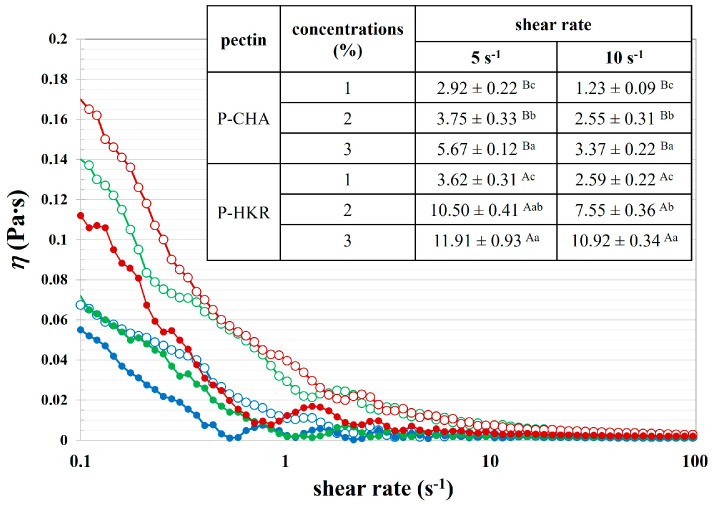
Apparent viscosity of P-CHA (closed symbols) and P-HKR (open symbols) at varying concentrations of 1% (●, ◯), 2 % (●, ◯), and 3 % (●, ◯). The inset table shows apparent viscosity at fixed shear rates (5 s^−1^ and 10 s^−1^) of P-CHA and P-HKR. Different capital superscripts indicate significant differences between means as affected by pectin sample (*p* ≤ 0.05), and lowercase superscripts indicate significant differences between means as affected by pectin concentration (*p* ≤ 0.05).

The functional properties of P-CHA and P-HKR were examined, with Figure 5 presenting the rheological properties of the pectin samples at varying concentrations. Rheological behavior is a crucial property, determining the feasibility for the application of pectin in food products. There was a decrease in the apparent viscosity with an increasing shear rate for the P-CHA and P-HKR solutions, indicating non-Newtonian pseudoplastic fluid behavior with a shear-thinning effect of the pectin samples [35]. With an increased shear rate, the pectin might rearrange to a more orderly molecular structure, leading to less interaction between the neighboring molecules and, thereby, decreasing viscosity [35]. This behavior was also found for the pectin extracted from apple peels [10] and fig peels [49]. Higher apparent viscosity was observed when the pectin concentration was increased, which is in accordance with other studies [10,49]. 

There was a higher apparent viscosity at a fixed shear rate for P-HKR (see inset table in Figure 5). This result might be interpreted due to the different molecular characteristics of the pectin sample. The greater degree of molecular branching with a higher DM previously suggested for P-HKR might allow for better interaction with the water molecules, thereby promoting a rheological controlling capability. Apparent viscosity is a crucial factor, indicating the thickening effect of pectin, thereby determining effective functional properties for the production of several foods. Pectin with a potent thickening effect might also possess bioactivity, such as the ability to control lipolysis, plasma, and cholesterol levels [10].

The emulsifying capacity of the pectin samples is reported in Table 3. Higher EAI was found for the emulsion stabilized by P-CHA, whereas stabilizing using P-HKR resulted in greater ESI and a lower creaming rate of the emulsion. The emulsifying activity of pectin could be expected due to the presence of the RG-I domain [41] and a residual proteinaceous component [47] in the pectin structure. The molecular characteristics of pectin, such as DM, composited monosaccharides, and molecular size, greatly affected the functional properties, involving the interfacial activity, emulsifying ability, foaming capacity, and bioactivity of pectin [4]. The present results implied better emulsifying formability with greater interfacial activity for P-CHA than for P-HKR. At a neutral pH, as was applied in the present conditions, interfacial adsorption of pectin was suggested to have loop and tail behavior, with pectin with greater molecular linearity being able to cover interfacial areas more effectively than ones with lower molecular linearity [50]. P-CHA had a lower DM than P-KHR, suggesting the greater molecular linearity of the former pectin, so effective interfacial absorbability might be supposed for P-CHA. With the higher degree of branching for P-HKR, the sugar side chains might obstruct pectin adsorption to the interfacial areas, resulting in lowering emulsion formability [51]. Nevertheless, the higher ESI and lower creaming rate for the P-HKR-based emulsion might be explained by the greater thickening effect of P-HKR, as previously suggested (Figure 5). Pectin with a high thickening effect could increase the viscosity of the aqueous phase, thereby promoting the colloidal stability of the emulsion [5].

The foaming property of the cannabis pectin was examined, with the results shown in Table 4. P-CHA possessed greater foam-forming ability, as implied by its higher FI. The lower FI of P-HKR might have been due to the greater thickening effect of the pectin, which affected the promotion of viscosity in the aqueous phase, so that the incorporation of air bubbles might have been harder, resulting in a reduced foam volume [47]. Foam is a colloidal system consisting of hydrophobic air bubbles dispersing in a hydrophilic liquid continuous phase. Therefore, interfacial capacity affected the foam-forming capacity of the pectin solution. It should be noted that the greater FI was in accordance with the greater EAI of P-CHA compared to P-HKR. Furthermore, the greater presence of phenolic residues might have enhanced the foaming capability of P-CHA by lowering the surface tension between the air–water phases [47]. An increased concentration of pectin led to improved FS, which was consistent with previous works reporting on the functional properties of the pectin extracted from other plants such as sunflower [47] and eggplant [23] residues. This behavior might be explained by the enhanced viscosity of the pectin solution at increased concentrations, as reported in Figure 5, because the thickening effect of the aqueous phase could promote the stability of the air bubbles in the foam matrix [23,47].

## 3. Materials and Methods

### 3.1. Materials

Two cultivars of *C. sativa*—Chalotte’s Angels (CHA) and Hang-Krarog (HKR)—were used as the raw material. The CHA and HKR cannabis cultivars were grown in the greenhouses of Kasetsart University located in the Bangkok and Sakhon Nakhon provinces (Thailand), respectively. The stems and roots of the cannabis trees were received after cultivation of the inflorescence and leaves. Tartaric acid and citric acid were sourced from KemAus (Cherrybrook, NSW, Australia). Gallic acid, tannic acid, *1,1*-diphenyl-*2*-picrylhydrazyl (DPPH), *2,2*′-azinobis (*3*-ethylbenzothiazoline-*6*-sulfonic acid) diammonium salt (ABTS), *6*-hydroxy-*2,5,7,8*-tetramethylchromane-*2*-carboxylic acid (Trolox), and *m*-hydroxybiphenyl were purchased from Sigma-Aldrich Chemical Co. (St. Louis, MO, USA). All the reagents were analytical grade.

### 3.2. Extraction of Pectin

First, the selected parts of the cannabis trees were pretreated by washing and drying (60 °C, 5 h) using a tray dryer (BWS-model; Frecon; Bangkok; Thailand). After grinding, the sample was mixed with 85% ethanol at a solid-to-liquid ratio of 1:7.5 at 70 °C for 20 min. Then, the alcohol-insoluble fraction (AIF) was collected after drying the mixture at ambient temperature overnight. The AIF powder was kept in a polyethylene bag at 4 °C for less than 3 months before use.

#### 3.2.1. Calcium Pectate Content Preliminary Test

First, the amount of pectin present in the selected parts of the cannabis trees was estimated as the calcium pectate content based on a gravimetric method described by Rangana [52], with a slight modification. The AIF powder from plant materials (20 g) was mixed with HCl solution (0.05 N, 200 mL) at a solid-to-liquid ratio of 1:10 *wt*/*v* at 85 °C for 120 min. After adjusting the volume to 250 mL with deionized (DI) water, the mixture was passed through filter paper that had been pretreated by washing with hot water and drying (102 °C for 120 min). The sample residue was neutralized using an aqueous NaOH solution (1 M) and allowed to stand at ambient temperature overnight. Then, the mixture was added with acetic acid solution (1 N, 20 mL) and CaCl_2_ solution (1 M, 10 mL), stirred (450 rpm) continuously at room temperature for 60 min, heated until boiling for 3 min, and then filtered through the previous filter paper. The residue was dried at 102 °C until it reached a constant weight. The pectate content was calculated using Equation (1).

(1) $$Calcium\;pectate(\%)=\frac{{Wt}_{residue}\times 250\times 100}{{Wt}_{sample}\times V_{sample}}$$

where Wt_residue_ and Wt_sample_ are the weights of calcium pectate residue after drying and weight of the AIF, respectively, and V_sample_ is the total volume of the neutralized residue added with acetic acid and CaCl_2_ solutions.

#### 3.2.2. Pectin Extraction 

Extraction of pectin from the selected parts of the cannabis trees was performed using tartaric and citric acids following the method of Koubala et al. [53], with some modification. First, the solutions of tartaric acid (2 M) and citric acid (2 M) were prepared. The AIF powder of the plant materials was mixed with the selected acid solution at a solid-to-liquid ratio of 1:10. Then, pectin extraction using dissimilar acid solutions was controlled by adjusting pH of the mixtures to 2.0 by adding a small aliquot of the corresponding acid solution. The extraction was performed at 80 °C for 2 h with continuous stirring. After the designated time, the solid part was removed by filtering through a cheesecloth. Then, a portion of the liquid part was added with double the volume of 95% ethanol solution, stirred for 30 min, and left to stand at 4 °C for 24 h. The precipitate was washed thrice with 70% ethanol and once with 95% ethanol, before centrifuging (4000 rpm, 10 min) at 4 °C. The precipitate was air-dried at 45 °C until the moisture content was less than 10%. The extraction yield was determined based on the mass ratio of the derived pectin to the AIF of the plant materials.

### 3.3. Characterization and Functional Property of Pectin

The effects of extracting the acid solutions on the properties of the derived pectin were observed based on measurements.

#### 3.3.1. Physiochemical Properties

- Galacturonic acid (GalA) content: The content of GalA was quantified using the *m*-hydroxybiphenyl method [54]. The aqueous solution of extracted pectin (1%, 1 mL) was reacted with sulfuric acid solution (12.5 mM, 5 mL), before heating at 80 °C for 10 min. After cooling to room temperature, *m*-hydroxybiphenyl solution (0.15%, 100 mL) was added to the sample. The mixture was incubated for 20 min before measuring the absorbance at 520 nm (UV-1900; Shimadzu Co., Ltd.; Kyoto, Japan). The GalA content of the pectin sample was quantified based on a standard curve of galacturonic acid (0–100 mg/L).
- Methoxyl content: The methoxyl content was estimated based on a titration method [55]. The extracted pectin (0.2 g) was mixed with ethanol (2 mL), before adding NaCl (0.5 g) and DI water (50 mL). After mixing, titration with a standard NaOH solution (0.1 M) was performed using phenolphthalein as an indicator. After neutralization, the sample was well mixed with the NaOH solution (12.5 mL) in a closed Erlenmeyer flask and left to stand at room temperature for 30 min. Then, HCl solution (0.1 N, 12.5 mL) was added to the sample and titrated with the NaOH solution (the used volume was recorded as V_NaOH_). The methoxyl content was calculated using Equation (2).
  (2) $$Methoxyl\;content\left(\%\right)=\frac{V_{NaOH}\times 31\times N_{NaOH}}{{Wt}_{sample}}\times 100$$
- Fourier-transform infrared spectrometry (FT-IR) study: The chemical characteristics of pectin were examined using FT-IR spectrometry (model Tensor 27 spectrometer; Bruker; Ettlingen, Germany). Pectin sample was mixed with KBr crystals at a weight ratio of 1:150, and the spectra were recorded in a range 4000–500 cm^−1^ with a resolution of 4 cm^−1^.
- Degree of methyl-esterification (DM): The DM was determined using the FTIR profile of the pectin sample. The specific bands at 1740 cm^−1^ and 1630 cm^−1^ corresponded to the esterified and free carboxyl groups, respectively [56], with the DM being calculated according to the peak areas at 1740 cm^−1^ ($A_{COO{CH}_{3}}$) and 1630 cm^−1^ (${A_{COOH}\;\mathrm{or}\;A}_{COONa}$) based on Equation (3) [57].
  (3) $$DM\left(\%\right)=\frac{A_{COO{CH}_{3}}}{A_{COO{CH}_{3}}+{A_{COOH}(\mathrm{or}\;A}_{COONa})}\times 100$$
- Differential scanning calorimetry (DSC) analysis: The thermal properties of the pectin were examined using DSC (204-F1; Phoenix; Netzsch, Germany). The sample was finely ground and immediately sealed in an aluminum crucible before heating from 30 °C to 300 °C at a rate of 10 °C/min. An empty aluminum crucible was used as a reference.
- Monosaccharide composition: Determination of the monosaccharide composition was conducted using the high-performance liquid chromatography technique described by Cheong et al. [58] and reported using the standard monosaccharides, consisting of arabinose (Ara), glucose (Glc), galactose (Gal), rhamnose (Rha), fucose (Fuc), mannose (Man), galacturonic acid (GalA), and glucuronic acid (GlcA).

#### 3.3.2. Antioxidant Activity

- DPPH radical scavenging ability: The DPPH scavenging ability of pectin was determined using the method of Brand-Williams [59], with some modifications. Briefly, the pectin solutions (0.5–5.0%, 0.5 mL) were mixed with the methanolic DPPH solution (2.5 μg/mL, 2.5 mL). The mixture was incubated at room temperature for 1 h before reading the absorbance at 515 nm. The DPPH radical scavenging ability of the sample was quantified based on Equation (4) [60].
  (4) $$Radical\;inhibition\;capacity\left(\%\right)=[1-\frac{{(Abs}_{sample}-{Abs}_{solv})}{{Abs}_{blank}}]\times 100$$
  where Abs_sample_ and Abs_solv_ are the absorbance of pectin solution after reacting with the radical solution and methanol, respectively; Abs_blank_ represents the absorbance of DI water reacted with the radical solution.
- ABTS radical scavenging ability: Firstly, solution of ABTS^●+^ radicals was prepared by mixing ABTS solution (7.4 mM) and K_2_S_2_O_8_ (2.6 mM) at room temperature for 12–16 h in the dark. Then, the ABTS^●+^ radical solution was diluted with methanol for absorbance of 1.1 ± 0.02 at 734 nm. The pectin solution (0.5–5%, 0.2 mL) was reacted with ABTS^●+^ radical solution (2.85 mL) and incubated in the dark at room temperature for 2 h, before reading the absorbance at 734 nm as per the method of Re et al. [61]. The ABTS radical scavenging ability of the sample was calculated based on Equation (4) [60].
- Reducing power: The reducibility of pectin was observed as per the method of Chen et al. [62], with some modifications. Briefly, the pectin solutions (0.5–5%, 2 mL) were added with potassium ferricyanide (1%, 2 mL) and allowed to incubate at 50 °C for 30 min. Then, trichloroacetic acid solution (10%, 2.5 mL) and freshly prepared ferric chloride solution (0.1%, 0.5 mL) were added to the mixture. After standing at 50 °C for 10 min, the absorbance at 700 nm was recorded to indicate the reducing power of the pectin. DI water was employed instead of the pectin solution as a blank for the measurement.

#### 3.3.3. Functional Properties of Pectin

- Rheological property: The rheological properties of the extracted pectin were examined using a rheometer (MCR 301; Anton Paar GmbH; Graz, Austria) as per the method of Liu et al. [35] with some modifications. The aqueous solutions of pectin were prepared at different concentrations (1, 2, and 3%, *wt/v*), and a steady shear measurement was performed over the range of 0.1–100 s^−1^ using a 40 mm plate geometry probe at 25 °C.
- Emulsifying property: First, pectin was dissolved in a phosphate buffer (10 mM, pH 7.0), before homogenizing with soybean oil at 20,000 rpm for 3 min to produce the emulsion with 0.1 oil fraction. Then, the emulsion dispersibility was estimated.
  - *Emulsion ability index (EAI) and emulsion stability index (ESI):* The emulsion was diluted with the buffer, before measuring the absorbance at 500 nm immediately (*A*_0_) and after 10 min (*A*_10_). EAI and ESI were calculated based on Equation 5 and Equation 6, respectively [63].
    (5) $$EAI({cm}^{2}/g)=[2\times 2.303\times A_{0}/0.25\times protein\;concentration]$$
    (6) $$ESI(min)=[A_{0}\times 10/(A_{0}-A_{10})]$$
  - *Creaming rate:* The prepared emulsion was centrifuged (4000 rpm, 5 min), and the creaming rate percentage was calculated based on the height of the separated serum phase to the height of the total emulsion before centrifugation.
- Foaming property: Aqueous solutions of pectin were prepared by dissolving the pectin in the buffer and homogenizing using a vortex for 3 min at room temperature. The volume of the initial pectin solution (V_pectin_) and the total volume of the mixture immediately after mixing (V_0_) and after 10 min (V_10_) were recorded to determine the foaming index (FI) and foaming stability (FS), as per Equations (7) and (8), respectively [64].
  (7) $$FI(\%)=[V_{0}-V_{pectin}]/V_{pectin}\times 100$$
  (8) $$\mathrm{FS}\;(\%)=[V_{10}-V_{pectin}]/V_{pectin}\times 100$$

### 3.4. Statistical Analysis

Samples were prepared independently in duplicate. Each measurement was conducted in triplicate, and the result was reported as mean ± standard deviation. Statistical differences between the means were analyzed by one-way analysis of variance and Tukey’s test at the 95% confidential level using SPSS for Windows software (version 12; Chicago, IL, USA).

## 4. Conclusions

Different plant cultivars and extracting solvents affected the characteristics of the extracted pectin, thereby producing different functional properties for the pectin. Extraction using tartaric acid provided a higher yield than citric acid. The pectin derived using tartaric acid had a higher GalA content, whereas the citric acid-assisted extraction produced pectin with a higher DM. P-CHA and P-HKR were both LMP, with DM values of ca. 30–35%. P-CHA had a higher GalA content and better thermal properties as well as the ability to promote emulsion and foam formation. On the other hand, P-HKR had a higher DM and superior thickening effect. P-CHA showed better free radical scavenging ability, whereas P-HKR showed better reducibility. This study suggests the feasible application of cannabis as a food additive with functional and bioactive properties. To elucidate its practical utilization, the functional properties in a food model and in vivo bioactivity of the pectin should be further studied.

## Figures and Tables

**Table 1:** Characteristics of pectin extracted from the stems of CHA and HKR using different extracting acid solutions.

Cannabis Cultivar	GalA Content (%) *		Methoxyl Content (%) **	
	Tartaric Acid	Citric Acid	Tartaric Acid	Citric Acid
CHA	92.4 ± 2.75	87.58 ± 1.26	5.91 ± 0.80	5.99 ± 0.72
HKR	87.42 ± 3.21	67.42 ± 3.25	6.45 ± 0.89	6.61 ± 1.29

* Extracting acid solution and cannabis cultivars had significant effect on GalA content of the extracted pectin (*p* ≤ 0.05). ** There was no difference between methoxyl content of the extracted pectin (*p* > 0.05).

**Table 2:** Molar ratio of composited monosaccharides of P-CHA and P-HKR.

Monosaccharides	P-CHA	P-HKR
Galacturonic acid (GalA)	2.3	2.0
Rhamnose (Rha)	0.3	0.4
Arabinose (Ara)	0.4	0.3
Glucuronic acid (GlcA)	1.2	1.7
Galactose (Gal)	0.3	0.2
Fucose (Fuc)	not detected	0.5
Glucose (Glu)	1.1	0.9

**Table 3:** Characteristics of P-CHA- and P-HKR-based emulsions.

Pectin	EAI (cm^2^/g)	ESI (min)	Creaming Rate (%)
P-CHA	15.02 ± 0.04 ^a^	21.40 ± 0.64 ^b^	18.42 ± 0.22 ^a^
P-HKR	14.46 ± 0.37 ^b^	25.20 ± 2.29 ^a^	17.52 ± 0.35 ^b^

Different lowercase superscripts in each column indicate significant differences between means (*p* ≤ 0.05).

**Table 4:** Foaming capacity of P-CHA and P-HKR at varying concentrations.

Pectin	Concentration (%)	FI (%)	FS (%)
P-CHA	1	6.62 ± 0.38 ^Ac^	5.63 ± 0.33 ^Ac^
	2	10.57 ± 0.23 ^Ab^	7.77 ± 0.28 ^Ab^
	3	12.29 ± 0.06 ^Aa^	11.71 ± 0.83 ^Aa^
P-HKR	1	6.76 ± 0.43 ^Bc^	5.04 ± 0.45 ^Bc^
	2	8.83 ± 0.41 ^Bb^	7.29 ± 0.40 ^Bb^
	3	11.08 ± 0.46 ^Ba^	9.26 ± 1.06 ^Ba^

In each column, different capital superscripts indicate significant differences between means as affected by pectin sample (*p* ≤ 0.05), while different lowercase superscripts indicate significant differences between means as affected by pectin concentration (*p* ≤ 0.05).

## Data Availability

Data are contained within the article.
